# Genomic characteristics and drug screening among organoids derived from non‐small cell lung cancer patients

**DOI:** 10.1111/1759-7714.13542

**Published:** 2020-07-07

**Authors:** Jing‐Hua Chen, Xiang‐Peng Chu, Jia‐Tao Zhang, Qiang Nie, Wen‐Fang Tang, Jian Su, Hong‐Hong Yan, Hong‐Ping Zheng, Ze‐Xin Chen, Xin Chen, Meng‐Meng Song, Xin Yi, Pan‐Song Li, Yan‐Fang Guan, Gang Li, Chu‐Xia Deng, Rafael Rosell, Yi‐Long Wu, Wen‐Zhao Zhong

**Affiliations:** ^1^ The Second School of Clinical Medicine Southern Medical University Guangzhou China; ^2^ Guangdong Lung Cancer Institute, Guangdong Provincial People's Hospital, Guangdong Key Laboratory of Lung Cancer Translational Medicine South China University of Technology & Guangdong Academy of Medical Sciences Guangzhou China; ^3^ Guangzhou Twelfth People's Hospital Guangzhou China; ^4^ Shantou University Medical College Shantou China; ^5^ Accurate International Biotechnology Co. Guangzhou China; ^6^ Geneplus‐Beijing Institute Beijing China; ^7^ University of Macau. Cancer Centre, Faculty of Health Sciences University of Macau Macau China; ^8^ Institut d'Investigació en Ciències de la Salut Germans Trias i Pujol Campus Can Ruti (Edifici Muntanya) Ctra. de Can Ruti Barcelona Spain

**Keywords:** Consistency analysis, drug screening, non‐small cell lung cancer, patient‐derived organoid, whole exome sequencing

## Abstract

**Background:**

Patient‐derived organoid (PDO) models are highly valuable and have potentially widespread clinical applications. However, limited information is available regarding organoid models of non‐small cell lung cancer (NSCLC). This study aimed to characterize the consistency between primary tumors in NSCLC and PDOs and to explore the applications of PDOs as preclinical models to understand and predict treatment response during lung cancer.

**Methods:**

Fresh tumor samples were harvested for organoid culture. Primary tumor samples and PDOs were analyzed via whole‐exome sequencing. Paired samples were subjected to immunohistochemical analysis. There were 26 antineoplastic drugs tested in the PDOs. Cell viability was assessed using the Cell Titer Glo assay 7–10 days after drug treatment. A heatmap of log‐transformed values of the half‐maximal inhibitory concentrations was generated on the basis of drug responses of PDOs through nonlinear regression (curve fit). A total of 12 patients (stages I–III) were enrolled, and 7 paired surgical tumors and PDOs were analyzed.

**Results:**

PDOs retained the histological and genetic characteristics of the primary tumors. The concordance between tumors and PDOs in mutations in the top 20 NSCLC‐related genes was >80% in five patients. Sample purity was significantly and positively associated with variant allele frequency (Pearson *r* = 0.82, *P* = 0.0005) and chromosome stability. The in vitro response to drug screening with PDOs revealed high correlation with the mutation profiles in the primary tumors.

**Conclusions:**

PDOs are highly credible models for detecting NSCLC and for prospective prediction of the treatment response for personalized precision medicine.

**Key points:**

Lung cancer organoid models could save precious time of drug testing on patients, and accurately select anticancer drugs according to the drug sensitivity results, so as to provide a powerful supplement and verification for the gene sequencing.

## Graphical abstract

Lung cancer organoid models could save the precious time of drug testing on patients, and accurately select anticancer drugs according to the drug sensitivity results, so as to provide a powerful supplement and verification for the gene sequencing.

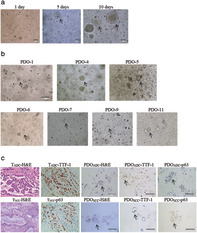

## Introduction

Notwithstanding the numerous efforts including smoking cessation, early detection and diagnosis, and combination and exploration of various strategies for antineoplastic therapy to reduce its morbidity and mortality rates, lung cancer remains the most prominent cancer among men and the second most prominent cancer among women and the leading cause of cancer‐related mortality.^1^ Rapid development of high‐throughput sequencing technologies has yielded various data, and numerous therapeutic targets for NSCLC have been reported. Therefore, drugs targeting specific therapeutic targets may be administered to lung cancer patients. However, treatment efficacy varies greatly owing to the heterogeneity of lung cancer, even with the same pathological tumor type. Therefore, current studies are focusing on establishing preclinical models more accruable and effective in identifying drug responses.

Classical preclinical models, including tumor and primary cell lines, and immunodeficient mouse models, are predominant in studies on cancers. Each of these models has advantages and drawbacks. Tumor and primary cell lines have the advantage of being easy to culture and amenable to modifications. However, this is a double‐edged sword. Numerous cell types can differentiate abnormally and lose their phenotype in a two‐dimensional (2D) culture environment.^2^ Furthermore, the drug sensitivity of cancer cells in a 2D environment is different from that in cells cultured in three‐dimensional (3D) cell culture systems.^3^ Although immunodeficient mouse models are tremendously helpful in understanding disease pathophysiology in vivo, model establishment is time‐consuming and may have a low success rate. Moreover, owing to their rodent origin, these models are unreliable for predicting human responses.^4^

Recent studies have focused on developing and utilizing new 3D organotypic models derived from embryonic stem cells, adult stem cells, induced pluripotent stem cells, and patient‐derived tissue fragments to study disease pathophysiology. Such models have the advantage of mimicking the tumor microenvironment in vivo and maintaining genomic stability upon long‐term culturing.^5^ Importantly, such 3D models have widespread applications in studies on cancers. They can be used for drug development, preclinical screening of anticancer drugs, and individualized assessment.^6^ Patient‐derived organoid (PDO) models of cancer, including PDOs of colorectal,^7^ gastrointestinal tract,^8^ prostate,^9^ pancreatic,^10^ liver,^11^ and breast^12^ cancers have been developed and applied in clinical studies. A recent study on lung cancer organoid models described the establishment of PDOs and drug testing.^13^ However, few studies have investigated the quality of these tumor organoid models.

Here, we assessed 3D organoid models of NSCLC and examined individual therapeutic strategies on the basis of drug screening. Furthermore, we analyzed tumor cell purity via whole‐exome sequencing (WES) and compared it with the patients' individual clinical data.

## Methods

### Patients and sample collection

Resected tumor specimens were harvested from lung cancer patients at Guangdong Provincial People's Hospital and transferred to RPMI medium (Gibco: Thermo Fisher Scientific, Waltham, MA, USA) supplemented with 50 ng/mL epidermal growth factor (EGF; PeproTech: Rocky Hill, NJ, USA), 10 μM Y‐27632 (Selleck), 5% fetal bovine serum (Gibco: Thermo Fisher Scientific, Waltham, MA, USA), and penicillin‐streptomycin, and transported directly to the laboratory. Adenocarcinoma (ADC) or squamous cell carcinoma (SCC) of the lung was diagnosed on the basis of pathological assessment of frozen slices. Tissue samples were used to establish organoid cultures and analyze the original tumors.

### Tissue dissociation and organoid culture

A total of 5–6 tumor fragments were generated using ophthalmic scissors, approximately 1 mm^3^, and separated into cold Hank's Balanced Salt Solution (HBSS) with antibiotics (Lonza, Basel, Switzerland) and transported to the laboratory on ice within one hour of resection from patients. After washing thrice with cold HBSS with antibiotics and sectioning with sterile blades, the samples were incubated with 0.001% DNase (Sigma‐Aldrich, MO, USA), 1 mg/mL collagenase/dispase (Roche, IN, USA), 200 U/mL penicillin, 200 mg/mL streptomycin, and 0.5 mg/mL amphotericin B (2% antibiotics, Sigma) in DMEM/F12 medium (Lonza) at 37°C for three hours with gentle agitation and intermittent resuspension. Thereafter, the digested tissue suspension was repeatedly triturated via pipetting and passed through a 70 μm filter. The strained suspension was centrifuged at 112 × *g* for three minutes, and red blood cells were lysed using lysis buffer (00443357, Invitrogen eBioscience) for five minutes. The pellet was resuspended in 100 μL MBM (serum‐free medium DMEM/F12; Lonza) supplemented with 20 ng/mL bFGF (Invitrogen, CA, USA), 50 ng/mL human EGF (Invitrogen), N2 (Invitrogen), B27 (Invitrogen), 10 μM ROCK inhibitor (Enzo Life Sciences, NY, USA), and 1% penicillin‐streptomycin (Gibco, OK, USA). Thereafter, 200 μL Matrigel (Corning, NY, USA) was added to 100 μL of the cell suspension for establishing organoids, and the resulting suspension was allowed to solidify on prewarmed 6‐well culture plates (Corning, NY, USA) at 37°C for 30 minutes. After gelation, 3 mL MBM was added to the well. The medium was changed every four days. Organoids were passaged every two weeks with the ratio of 1:2 or 1:3. The procedure for organoid passaging was modified from the methods previously described. Briefly, organoids were harvested by incubating with cold PBS for one hour at 4°C and dissociated using 1× TrypLe (Gibco). The dissociated organoids were then mixed in MBM + Matrigel (1:3 ratio) and reseeded in a Petri dish, followed by the addition of medium after gelling. Early passage organoids (<3 passages) were frozen in liquid nitrogen for further investigation.

### Organoid drug response assay

The primary organoids cultured over two weeks were harvested and dissociated using 1× TrypLe (Gibco). The dissociated organoids were mixed in MBM + Matrigel (1:3 ratio) and seeded onto 384 well white plates. After gelation, 30 μL MBM was added to each well. The organoids were cultured for 48 hours. The average diameter of organoids was set at 50 μm as the minimum requirement for drug screening. Thereafter, a dilution series of each compound (50, 10, 2, 0.4, 0.08, and 0.016 μM) was dispensed using liquid‐handling robotics, and cell viability was assayed using CellTiter‐Glo (Promega) after four days of drug incubation. The plates were agitated for 30 minutes at room temperature prior to measuring luminescence. Half‐maximal inhibitory concentration (IC50) values were determined using GraphPad Prism 7.0 (GraphPad Software, La Jolla, CA, USA).

### Histology and immunostaining

Organoids and their corresponding parental tumors were fixed in 4% paraformaldehyde, followed by paraffin embedding, sectioning, deparaffinization, dehydration, and hematoxylin‐eosin staining. Immunohistochemistry (IHC) was performed using antibodies targeting thyroid transcription factor (TTF‐1, clone 8G7G3/1, Ready‐to‐Use) and p63 (clone DAK‐p63, Ready‐to‐Use), and then 3,3′‐Diaminobenzidine was used for color development. Images were acquired on an OLYMPUS IX73 microscope (Olympus, Tokyo, Japan).

### Whole‐exome sequencing

Formalin‐fixed, paraffin‐embedded (FFPE) samples were extracted using the QIAamp DNA FFPE Tissue Kit (Qiagen, Hilden, Germany). DNA concentration from peripheral blood lymphocytes and tissues was measured using a Qubit fluorometer (Invitrogen, Carlsbad, CA USA) and the Qubit dsDNA BR Assay Kit.

Library construction was performed using the Illumina TruSeq DNA Library Preparation Kit (Illumina, San Diego, CA, USA) with 1 μg of DNA sheared using an ultrasonicator to generate fragments of maximum 200–250 bp, followed by end repair. Tailing and ligation to the Illumina‐indexed adapters were performed in accordance with the standard library construction protocol. Libraries were hybridized to custom‐designed biotinylated oligonucleotide probes (Roche NimbleGen, Madison, WI, USA) encompassing approximately 64 Mbp. DNA sequencing was performed using the BGI‐seq 500 Sequencing System with 2 × 100 bp paired‐end reads.

### Identification of somatic mutations and copy number variations

Adapter sequences and low‐quality reads were eliminated from the raw data. BWA‐MEM^14^ (version 0.7.12‐r1039) was used to align the clean reads to the reference human genome (hg37). Single nucleotide variants (SNVs) and small insertions and deletions were called using Sentieon software (https://www.sentieon.com). A mutation was identified as a candidate somatic mutation only when (i) the mutation had a variant allele fraction (VAF) ≥ 5% and at least five high‐quality reads (Phred score ≥ 30, mapping quality ≥13, and without paired‐end reads bias) supporting the particular base; (ii) the mutation frequency was ≥5‐fold in the tumor tissue rather than in the normal tissue; and (iii) the mutation was not present in >1% of the population in the 1000 Genomes Project or the Single Nucleotide Polymorphism databases.^15^, ^16^

Candidate mutations were annotated to genes using the VEP^17^ software to identify the mutated protein‐coding position and filtered to exclude intronic and silent changes, while retaining missense, nonsense, frameshift, spans, splicing, cds‐del, cds‐ins, stop‐gain, and stop‐loss mutations.

Somatic copy number variation (CNV), tumor purity, and ploidy were assessed from WES data, using FACETS.^18^ Thresholds of 0.75 and 1.25 were used to delineate the cutoff for deletions and amplifications, respectively.

### Mutation analysis

We first determined the correlations among organoids, tumors, and peripheral blood of seven patients on the basis of germline mutations. All samples were clustered by 1‐correlation distance using complete linkage for hierarchical clustering. The most prevalent mutated genes in lung cancer were obtained from cBioPortal for Cancer Genomics (http://www.cbioportal.org).

Mutation signatures and point mutation type distributions in tissue and PDOs per patient were determined and visualized using the Mutational Patterns (http://bioconductor.org/packages/release/bioc/html/MutationalPatterns.html) R package. The correlation of VAFs between the tumor and PDO and that between tumor purity and median VAF in the tumor and PDO were determined with the lm function, using the native stats package. The Ggpubr (https://cran.r-project.org/web/packages/ggpubr/index.html) R package was used to compare the VAF of common mutations between the tumor and PDO for each patient. *P*‐values were calculated using paired student's *t*‐test (*P* < .05 was considered statistically significant).

### Ethics approval and consent to participate

The study methodologies conform to the standards set by the Declaration of Helsinki. Ethical approval was obtained from the Research Ethics Committee of Guangdong Provincial People's Hospital, Guangdong Academy of Medical Sciences (Approval No. GDREC2016175H). Informed consent was obtained from all patients prior to tissue acquisition.

## Results

### Establishment of PDO models for NSCLC

To establish a preclinical model closely related to the structure and function of the human body, we carried out 3D culturing of established PDOs from NSCLC patients. Seven patients with pathological stage I–III NSCLC were enrolled. Fresh tumor specimens were intraoperatively harvested (Table 1). Six ADCs (86%) and one SCC (14%) were pathologically diagnosed.

**Table 1:** Baseline characteristics of the patients

Characteristic	Patients (*n* = 7)
Age	
Mean ± SD	63.9 ± 5.5
Median (range, year)	66 (56–69)
Sex (%)	
Female	1 (14)
Male	6( 86)
Histologic diagnosis (%)	
Adenocarcinoma	6 (86)
Squamous cell carcinoma	1 (14)
Clinical disease stage	
I	3 (43)
II	3 (43)
III	1 (14)
Smoking status ‐ No. (%)	
Never	2 (29)
Former or current	5 (71)

SD, standard deviation.

**Figure 1:**
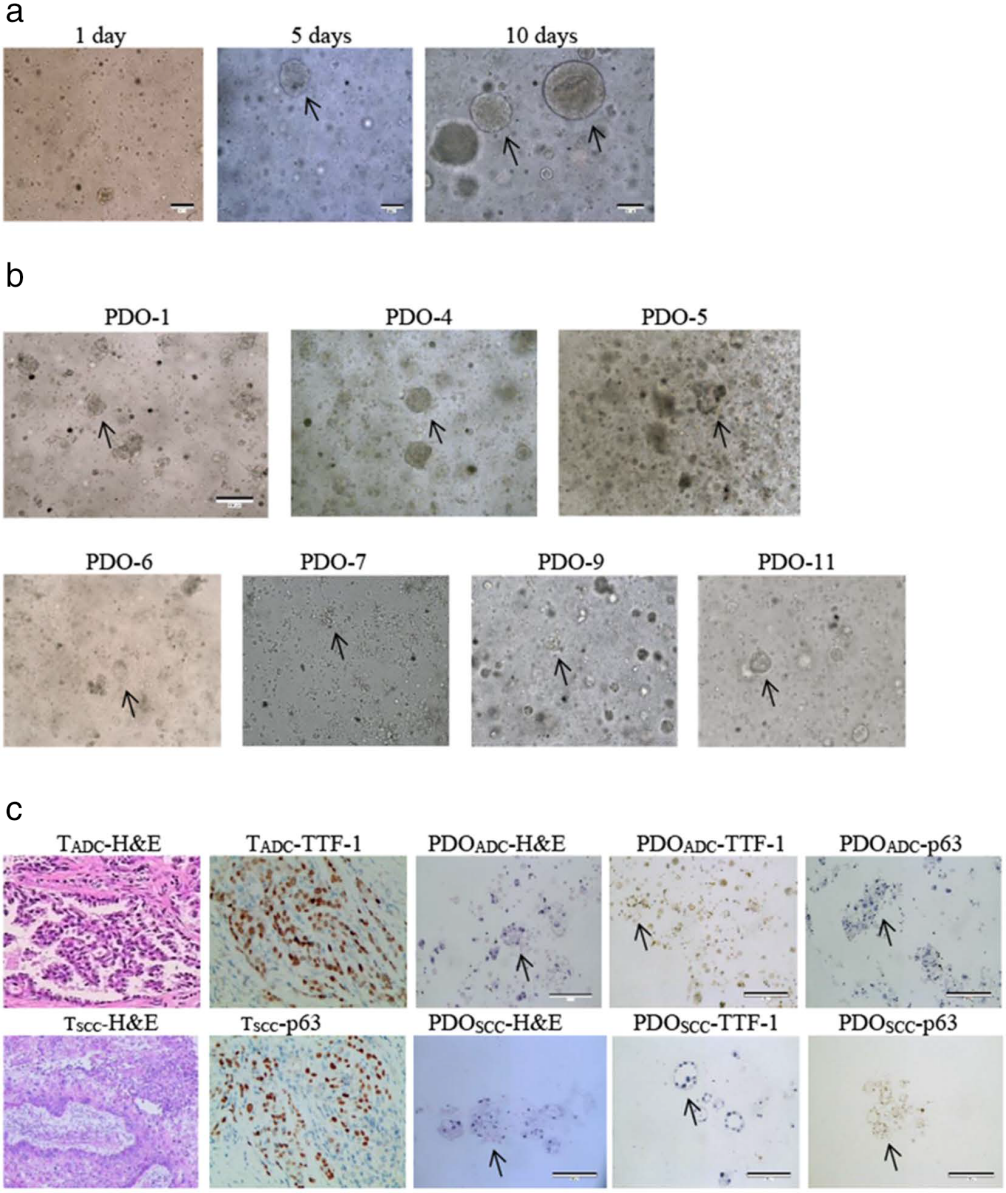
Patient‐derived organoids (PDOs) of NSCLC recapitulate the parental tumor. (**a**) The PDO phenotype was observed with a bright field microscope, as shown by the black arrows, after one week of culture. Scale bar, 100 μm. (**b**) Morphological changes of PDOs after the stated number of days in culture. P4 is squamous cell carcinoma; all others are adenocarcinoma. Scale bar, 100 μm. (**c**) HE‐stained and IHC images of PDOs and their parental tumor tissues. ADC marker TTF‐1 and SCC marker p63 were expressed in tumor tissues and corresponding organoids, respectively. ADC, adenocarcinoma; HE, hematoxylin‐eosin staining; IHC, immunohistochemistry; NSCLC, non‐small cell lung cancer; PDO, patient‐derived organoid; SCC, squamous cell carcinoma; T, tumor; TTF‐1, thyroid transcription factor‐1.

Over time, the PDOs gradually aggregated from the original monolayer into 3D cell clusters and became enlarged (Fig ). The established PDO models were cultured for at least two to three weeks (Fig 1b). Thereafter, we investigated whether the 3D PDOs retained the histological features of the original tumor tissues. Paired samples were histologically and cytologically examined via conventional histological methods after fixation with absolute ethanol. Paraffin sections were serially sliced into 5 μm‐thick sections and stained with hematoxylin‐eosin. Histomorphology results showed that most PDOs, either ADC or SCC, showed solid masses and that some of them had cystic structures, with irregular cell size and nuclear shape. However, it is impossible to distinguish between tumor organoids and normal airway organoids based on histomorphological features only.^19^ Therefore, we performed IHC staining to analyze the molecular characteristics of PDOs. IHC features of PDOs derived from ADCs and SCCs were highly concordant with those of their primary tumors. Furthermore, the p63 expression profile of SCC organoids did not show a polarized pattern, suggesting that the SCC organoids were composed of tumor cells rather than normal airway cells (Fig 1c).

### Conserved genotypic features between original tumor tissues and organoids

**Figure 2:**
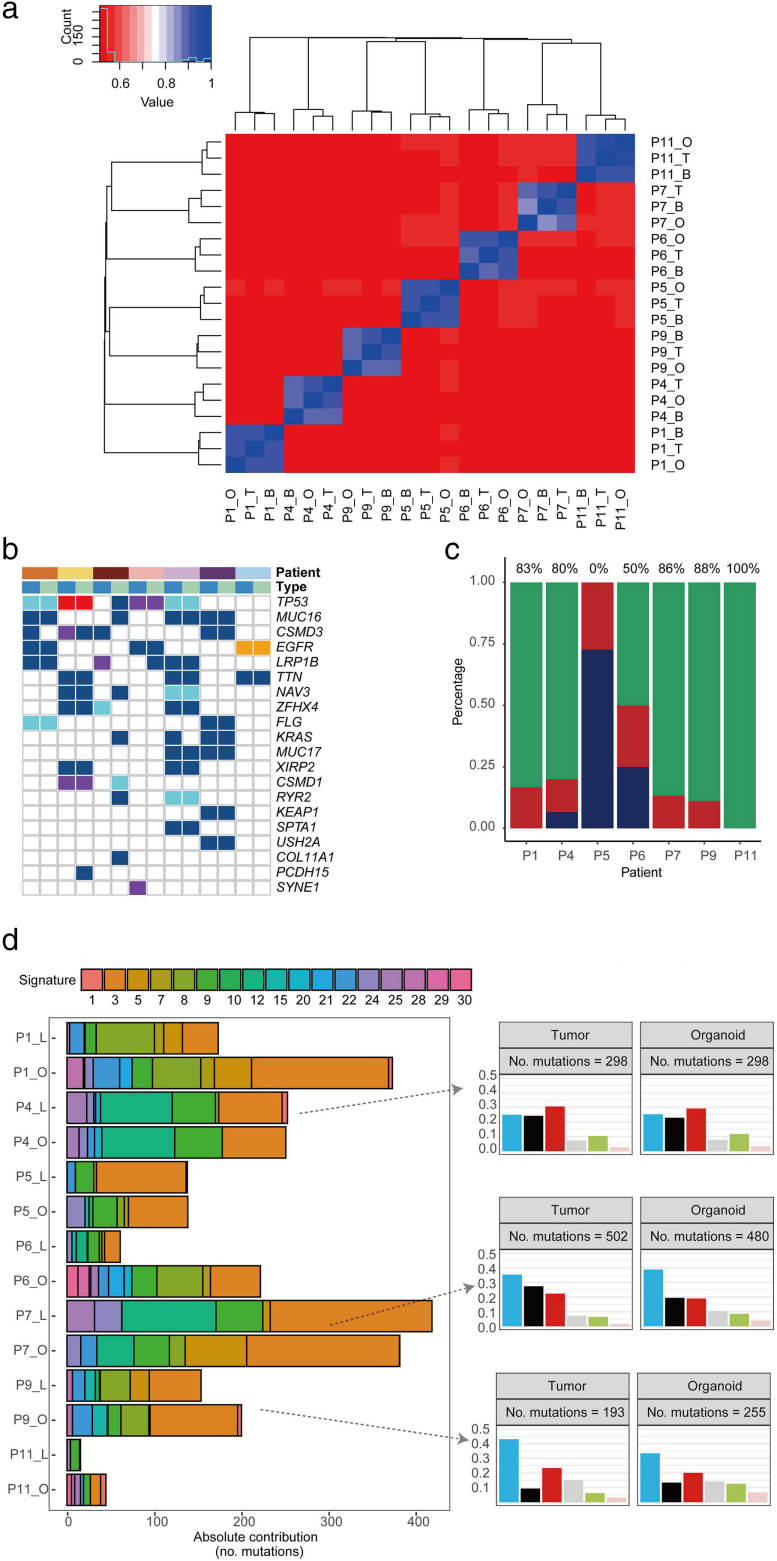
Genotypic features of the paired samples. (**a**) Heat map of the germline mutations in the peripheral blood, organoids, and tumor tissues of each patient. (**b**) Mutations detected in paired tissues (tumor and organoid) on the basis of gene mutations in lung cancer (top 20 genes are shown) Patient (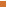) P1, (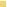) P4, (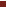) P5, (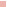) P6, (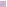) P7, (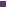) P9, and (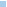) P11; Mutation (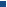) Missense, (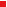) Nonsense, (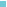) Frame Shift, (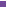) Splicing, (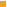) In Frame Indel; Type (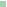) Tumor, and (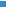) Organoid. (**c**) Concordance of somatic mutations detected in the organoid and corresponding tumor tissue (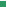) Concordant, (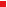) Organoid only, and (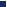) Tumor only. (**d**) Mutation signature distributions of organoid lines and tumor tissues in each patient. Signature types are displayed in the right panel. C > A, C > G, and C > T were the most common point mutations in our paired samples Point mutation type (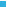) C>A, (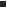) C>G, (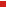) C>T, (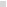) T>A, (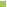) T>C, and (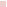) T>G.

To determine whether the PDOs maintained the mutational landscape of their corresponding parent tumor samples, we performed WES to analyze and compare the genomic characteristics of the original tumor tissues and PDOs.^20^, ^21^ Genomic DNA was extracted from paired tumors, organoids, and matched peripheral blood lymphocytes to identify tumor‐ and lung cancer‐related somatic mutations. Germline mutations were detected to eliminate potential cross‐contamination between samples (Fig 2a). The average sequencing depth of all tumors and organoids was approximately 249 (Table S1). In total, 1625 somatic mutations were detected in tumors and PDOs. Based on the top 20 prevalent lung cancer‐related genes, the median consistency of SNVs between tumors and organoids was over 80%, except for P5, which displayed 0% concordance; P11 displayed 100% concordance with the paired sample (Fig 2b). *TP53* (71%), epidermal growth factor receptor (*EGFR)* (43%), and *KRAS* (29%) were the most frequently mutated genes in this cohort, with frequencies of 57%, 43%, and 29%, respectively, in the matched PDOs (Fig 2c).

Furthermore, we quantified the relevant contributions of 30 COSMIC mutational signatures for each patient (Fig 2d). As previously reported, owing to tumor heterogeneity, most individual cancer genomes exhibit more than one mutational signature, and numerous different combinations of features were observed in our paired samples. The contribution patterns of individual cancer samples significantly differed between signatures. Therefore, we analyzed the type distribution of point mutations in each sample. In the organoid lines, most mutation loads and types of point mutation were conserved from the original samples. In terms of base substitutions in the tumor samples and organoids, P4, P7, and P9 displayed increased C > A, C > G, and C > T mutations, respectively.

In general, lung cancer organoids could recapitulate the diverse genomic landscape, including SNVs, the mutational load, and signatures of their original tumors, preserving the heterogeneity of various oncogenic mutations.

### Association between genomic characteristics and tumor purity

**Figure 3:**
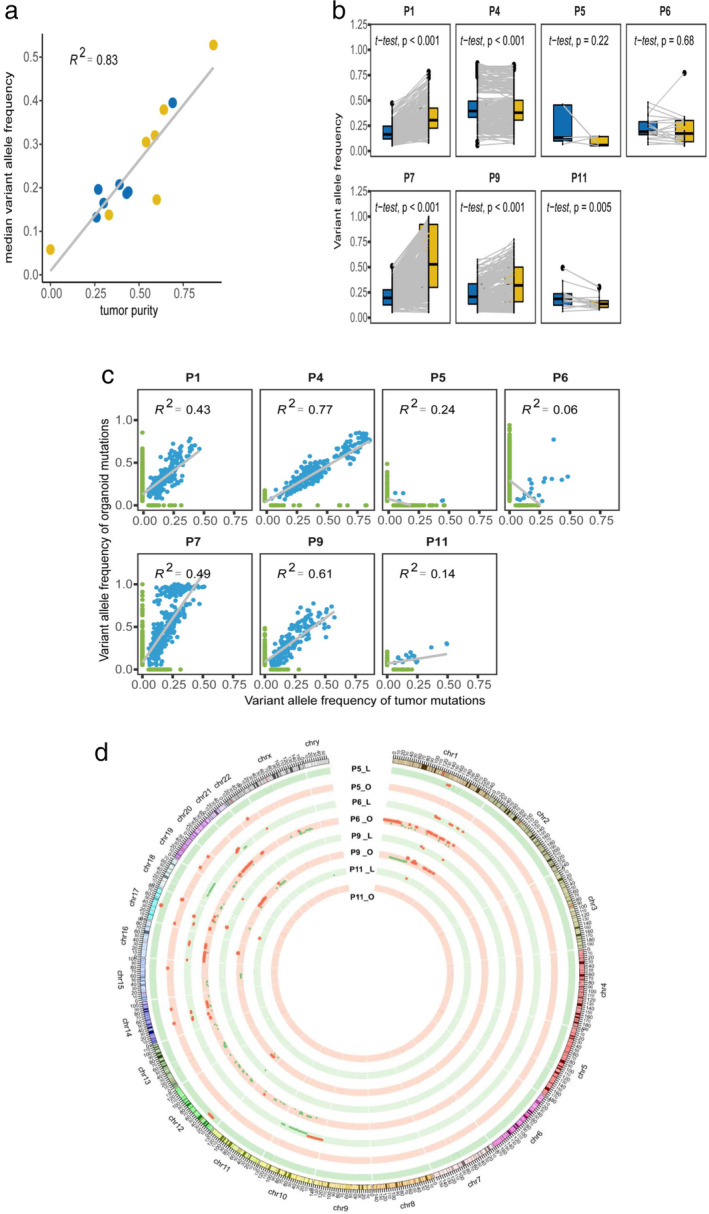
The association between tumor cell purity and VAF. (**a**) Correlation between the purity of the organoids and corresponding tumor samples and the median VAF (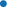) Tumor, and (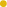) Organoid. (**b**) Comparison of VAF distributions of common mutations in organoids and tumor tissues of each patient (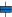) Tumor, and (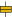) Organoid. (**c**) Correlation analysis of total mutations with VAF in organoids and tumor tissues in each patient. (**d**) Circos analysis of paired samples in the chromosomal context of PDOs. The outer layer represents the chromosome location; inner rings, CNVs at different chromosome loci. Red and green colors represent gains and losses in DNA copy number, respectively. CNV, copy number variation; VAF, variant allele frequency.

We estimated tumor purity using the FACETS software. Tumor purity was significantly and positively associated with the VAF (Spearman r = 0.83, *P* < .001) (Fig 3a). To further evaluate the VAF distribution between tumor samples and PDOs, we compared shared mutation VAFs among the tumors of different patients and PDOs and found that the VAFs of the organoids were generally higher than those of the original tumors, except for P5 and P11 (Fig 3b). Furthermore, we analyzed the association between the VAFs of tumors and organoid mutations. Interestingly, except in P5, P6, and P11, in all other tumor tissues and PDOs, VAFs of tumors were positively correlated with those of organoids (Fig 3c). These findings indicate that tumor cell proliferation might gradually decrease during culturing, whereas that of other cells might increase.

Furthermore, we also observed gains and losses of DNA copy numbers in the paired samples. The tumor cells in PDOs displayed higher purity and more gains or losses of CNVs than did the original tumor samples (Fig 3d). However, these findings were not obtained with P5 and P11.

### Prediction of the efficacy of individualized treatment through drug screening

**Figure 4:**
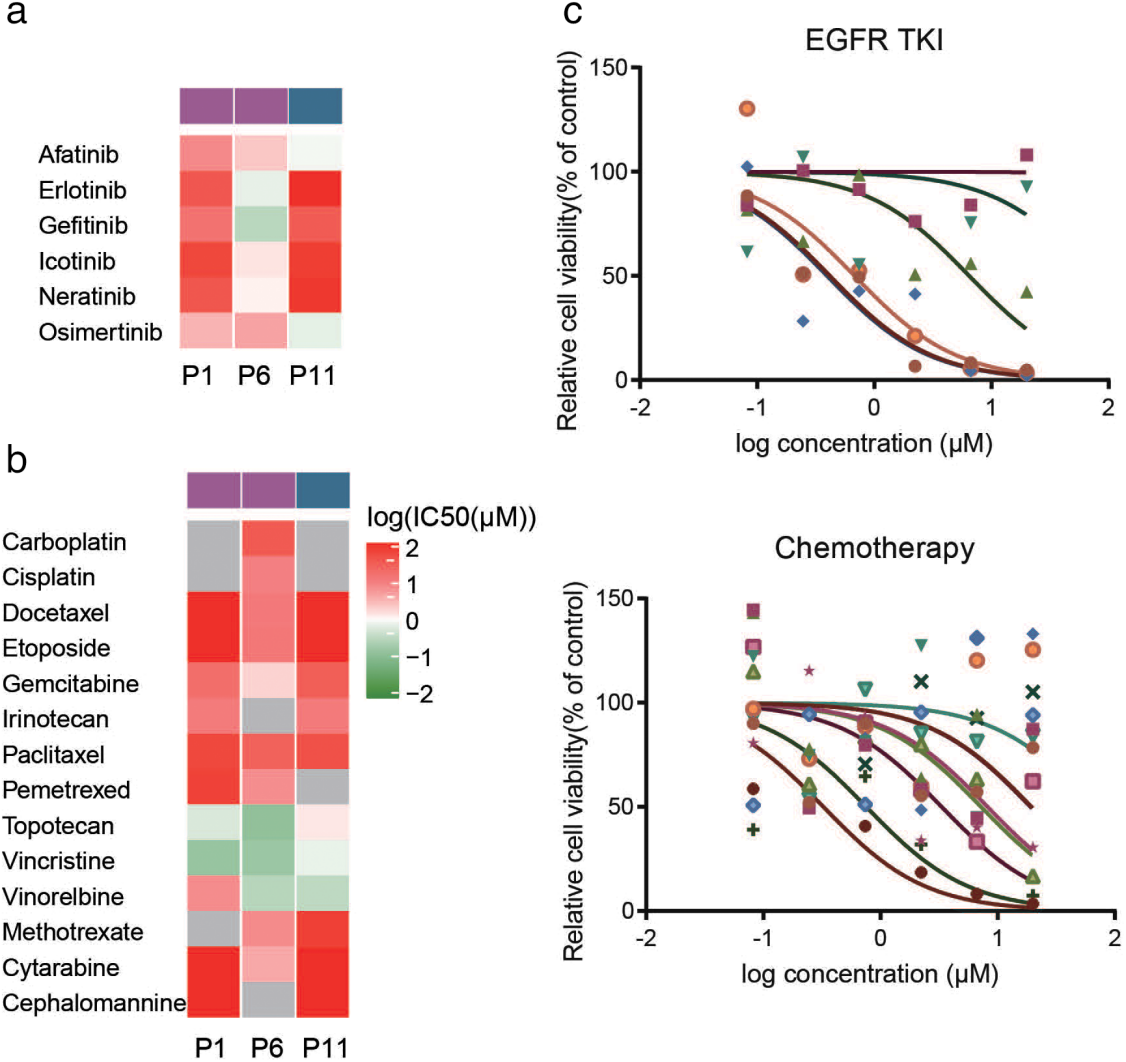
Heat map of drug response in Pptient‐derived organoids (PDOs). (**a**) The sensitivity to EGFR‐TKIs was analyzed in P1, P6, and P11, which harbored *EGFR* mutations (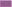) EGFR L858R, and (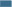) EGFR EX20ins. (**b**) The chemotherapeutic sensitivity was analyzed in P1, P6, and P11 (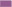) EGFR L858R, and (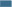) EGFR EX20ins. (**c**) Drug sensitivity screening was performed in P9, which harbored a *KRAS* mutation, using EGFR‐TKIs (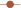) Afatinib, (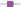) Erlotinib, (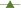) Gefitinib, (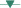) Icotinib, (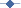) Neratinib, and (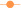) Osimertinib and chemotherapy drugs (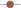) Methotrexate, (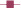) Cytarabine, (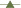) Cisplatin, (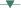) Carboplatin, (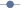) Docetaxel, (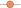) Etoposide, (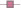) Gemcitabine, (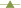) Irinotecan, (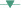) Paclitaxel, (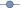) Pemetrexed, (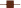) Topotecan, (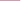) Vincristine, (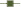) Vinorelbine, and (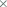) Cephalomannine. EGFR‐TKI, epidermal growth factor receptor tyrosine kinase inhibitor; PDO, patient‐derived organoid.

To evaluate the drug response of PDOs, 26 therapeutic agents, including chemotherapy and targeted drugs, selected on the basis of the treatment of NSCLC in accordance with the NCCN guidelines, were used to test the seven PDOs. IC50s and dose–response curves reflected the efficacy of each therapeutic agent (Fig 1 SuppInfo). The responses to these therapeutic agents in the PDOs, which partially correlated with their mutation profiles, revealed outstanding similarities and differences between the tumor and its corresponding PDO (Table S2). For example, both tumor samples and PDOs harbored driver mutations in four patients (*EGFR* L858R, *n* = 2; *EGFR* Ex20 ins, *n* = 1; and *KRAS* G12C, *n* = 1). Of the two PDOs harboring the *EGFR* L858R mutation, PDO‐6 displayed the most significant response to gefitinib. In contrast, PDO‐1 displayed gefitinib resistance but a significant response to osimertinib. The matched tumor tissue overexpressed c‐MET, indicating a mechanism underlying gefitinib resistance (Fig 4a). PDO‐11, harboring an *EGFR* exon 20 insertion mutation, was also resistant to gefitinib but showed a significant response to osimertinib and chemotherapy (Fig 4a,b). These results are concurrent with those of a previous report wherein tumors harboring this mutation displayed a similar treatment response.^22^, ^23^

*KRAS* mutations are present in approximately 30% of Caucasian individuals and 10% of East Asian individuals with lung adenocarcinoma.^24^, ^25^ They are more common among smokers (30%) than among non‐smokers (10%).^26^ Numerous previous studies have reported intrinsic resistance to erlotinib and gefitinib among NSCLC patients harboring *KRAS* mutations.^27^, ^28^ However, numerous recent studies have reported that irreversible tyrosine kinase inhibitors (TKIs), such as afatinib and neratinib, downregulate ERBB2 and ERBB3 and improve the efficacy of TKIs.^29^, ^30^, ^31^ Interestingly, our results validated the effectiveness of the pan‐ERBB inhibitor afatinib in P9, which harbored a *KRAS* G12C mutation (Fig 4c). In summary, the results of targeted therapeutic sensitivities in PDO pharmacogenomics may complement precision medicine approaches.

## Discussion

The 3D organoid model has introduced a new era of preclinical research models that rectify the deficiencies in 2D cell cultures and PDX mouse models. PDOs can be used to assess not only disease etiology but also disease occurrence and development. Owing to their short modeling time and high growth rate, PDOs are potentially time‐saving and cost‐effective, especially among cancer patients. They can also accelerate the pace for studies on cancers and individualized precision medicine.

In this study, we established seven NSCLC organoid models derived from patient tumor surgical tissue, which matched the original tissue specimens and faithfully reflected the histological and genomic spectrum of NSCLC. However, the organoid culture and WES analysis revealed that the growth status of each lung cancer organoid model was not fully consistent. There are two potential reasons for this inconsistency: the first is the different skill levels of the sample collectors, and the second is intrinsic tumor heterogeneity.^32^, ^33^ Since in this study, sample collection was performed by a single professionally trained individual, it can be reasonably assumed that the second reason is more likely. The complex microenvironment in the 3D organoids comprises different types of cells growing together with an interdependent and competitive relationship.^34^ The proportion of these cells in the original tumor tissue differed among different samples. However, it is still a difficult task to identify the specific proportion of dozens of cell types in the samples. Tumor purity and genetic features are reportedly associated with each other.^35^, ^36^ Recently, Dijkstra *et al*. established over 70 lung cancer organoids but only 17% of them were pure tumor organoids, and most of the organoids presented features of normal airway organoids.^19^ Interestingly, our study showed that tumor cell purity of the PDOs had a high concordance with those in the original tissues, indicating that the accuracy of sampling could be critical for the success of organoid establishment. Moreover, we analyzed the association between tumor cell purity of the PDOs and corresponding VAFs and confirmed a significant linear relationship between tumor cell purity and VAF. Tumor cell purity may be an appropriate indicator of the growth status of tumors and other cells in organoids. Tumor‐derived organoids contain tumor stem cells, which lead to tumor cell enrichment and the expansion of genetic information. Furthermore, tumor‐derived organoids reportedly have a high percentage of mitotic failure and subsequent cell death.^6^ This study showed that the lower the tumor cell purity (PDO‐5 and PDO‐11), the less prominent the two aforementioned effects. Accordingly, we speculated that the growth discrepancy in organoids was an intrinsic tumor characteristic. PDO models could, thus, truly preserve the heterogeneity of the original tumor tissue.

The many mutational features within cancer genomes suggest a potential mechanism underlying carcinogenesis. Understanding the etiology of these mutational patterns has potential implications for disease prevention and treatment. The most common point mutations herein were C > A, C > G, and C > T mutations. The C > A pattern, which usually results from bulky DNA adducts produced by polycyclic hydrocarbons in tobacco smoke and eliminated through transcription coupled with nucleotide excision repair, is observed in ADC, SCC, and small‐cell carcinoma. ^37^ Conversely, C > T and C > G mutations in TpCpN trinucleotides result from enzymatic damage, and are the most widespread mutation signatures in human cancers. These mutations are caused by off‐target modifications and DNA base excision repair by the APOBEC family of proteins.^38^ Although CNVs were fewer than SNVs herein, the proportion of CNVs encompassing human chromosomes was markedly higher. CNVs are generated through various mechanisms, and the mutation rate is markedly higher than that of SNVs.^39^, ^40^ Furthermore, CNVs have a greater impact on genomic evolution than SNVs. Most CNVs have been subjected to natural selection and genetic drift, and are key factors in studies on population history, migration, and evolution.^41^ This study showed that the changes in CNVs at different chromosomal loci could truly reflect the corresponding clinical features and genomic and biological characteristics. Recently, similar studies have also been published.^13^, ^42^ These studies also focused on the culture, validation and drug response. However, they emphasized the long‐term culture and passaging for building up a preclinical model. In our study, we focused on early to intermediate phase of NSCLC patients, then used the fresh organoid for validation and drug screening. The purpose of our study was to use an organoid model as a diagnostic tool to predict the clinical response of targeted therapy, so early passaged organoid was used. However, the small size of our cohort constituted a study limitation, and further large‐scale analyses using different methods are required to validate these findings regarding the genomic stability of the organoids and their prognostic potential and utility in studies on treatment responses.

Cancer genomes have provided remarkable insights into the molecular features of carcinomas. However, numerous drugs antagonize tumors with specific mutation types. The selection of a precise treatment for patients from multiple drugs is challenging. A promising advantage of PDO models is the ability to predict possible effective drugs for each individual patient. Here, we report a correlation between drug sensitivities and the mutation profile of PDOs. *EGFR* mutations, primarily occurring in exons 18 to 21, are some of the most frequent mutations in patients with lung adenocarcinoma. Exon 19 deletions and exon 21 L858R point mutations are considered to confer a favorable treatment response to first‐generation TKI therapy. However, uncommon *EGFR* mutations including the exon 20 insertion mutation (clustered between codons 767 and 774) account for 4%–10% of all *EGFR* mutations.^43^, ^44^ Tumors harboring *EGFR* exon 20 insertion mutations are resistant to first‐generation TKIs and sensitive to third‐generation EGFR‐TKIs.^22^ The present drug susceptibility test, which harnessed the genomic landscape to assess the tumor response, revealed that PDO‐11 was sensitive to the targeted drug osimertinib and the chemotherapeutic drug vinorelbine. Importantly, this may not be revealed drug sensitivity data by the routine genetic testing using tissue or blood samples from patients, which can only suggest a class of drugs that may be effective (and not specific drugs within a class – note that gefitinib and osimertinib are both EGFR inhibitors). This is the greatest advantage of PDOs, which will be more precise indicators of the appropriate treatments.

In summary, the PDO models of NSCLC established herein served as reliable in vitro cancer models that recapitulated the histological and genetic characteristics of their parental tumors. PDO models have advantages including a short timescale from establishment and the ability to combine genetic analyses with drug screening. Therefore, organoids could be a complementary method to help clinicians determine the most beneficial drug for individual patients. Over the next decade, studies are required to focus on appropriate methods of organoid culture to improve its efficacy and feasibility and to truly reflect the intratumor heterogeneity, since this would accelerate the progress and development of individual precision medicine.

## Disclosure

The authors declare that they have no competing interests.

## Supporting information


**Table S1** The average sequencing depth of all tumors and organoids.
